# Supplementary figures and images for: Bifidobacterium breve Alleviates DSS-Induced Colitis in Mice by Maintaining the Mucosal and Epithelial Barriers and Modulating Gut Microbes
Source: Nutrients. 2022 Sep 6;14(18):3671. doi: 10.3390/nu14183671 (PMC9503522; doi:10.3390/nu14183671)

## Supplementary Material

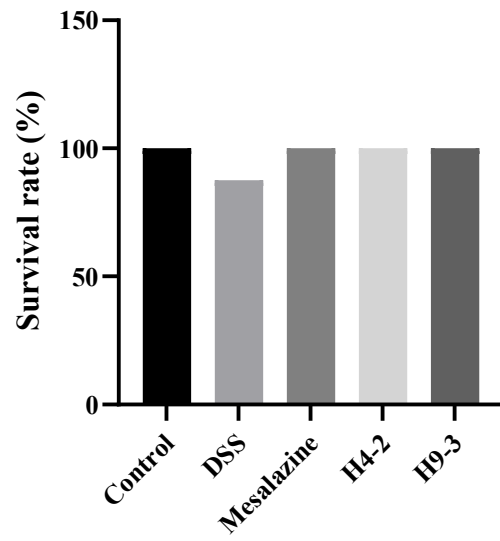

**Figure S1.** Percentage survival rate of C57BL/6J mice.

Supplement: Supplementary file 1 [file nutrients-14-03671-s001.zip › nutrients-1874332-supplementary.pdf]
